# Web-Based Training Program Using Cognitive Behavioral Therapy to Enhance Cognitive Flexibility and Alleviate Psychological Distress Among Schoolteachers: Pilot Randomized Controlled Trial

**DOI:** 10.2196/resprot.8541

**Published:** 2018-01-26

**Authors:** Satoru Oishi, Takeya Takizawa, Naoki Kamata, Shingo Miyaji, Katsutoshi Tanaka, Hitoshi Miyaoka

**Affiliations:** ^1^ Department of Psychiatry Kitasato University School of Medicine Sagamihara Japan; ^2^ Department of Occupational Mental Health Graduate School of Medical Sciences Kitasato University Sagamihara Japan

**Keywords:** school teachers, education, cognitive therapy, randomized controlled trial

## Abstract

**Background:**

Schoolteachers are known to be faced with various stresses in their work. A simple, less onerous, and effective intervention technique that can enhance the stress management skills, particularly, cognitive flexibility, of schoolteachers is needed.

**Objective:**

This study aimed to determine whether stress management training using a Web-based cognitive behavioral therapy (CBT) program is effective for enhancing the cognitive flexibility of schoolteachers and alleviating their subjective distress.

**Methods:**

This study was conducted in a random controlled design covering public elementary schoolteachers. Teachers allocated to the intervention group received 120 min of group education and completed homework using a Web-based CBT program that lasted for 3 months. The items of outcome evaluation were cognitive flexibility and subjective distress, and the efficacy of intervention was evaluated at 3 months after intervention.

**Results:**

A total of 240 participants were randomly allocated to the intervention group (120 individuals) and the control group (120 individuals). On the basis of the principle of intention to treat, the intervention group and the control group were compared regarding the amount of change from before intervention to after intervention, using a general linear model. Scores of cognitive flexibility and subjective distress were significantly more improved in the intervention group than in the control group.

**Conclusions:**

The results of this study suggest that simple stress management training using a Web-based CBT program in elementary schoolteachers enhances cognitive flexibility and alleviates subjective distress.

## Introduction

### Stress of Schoolteachers

It is known that schoolteachers are faced with much stress. It is therefore important to seek measures to enhance the stress management skills of schoolteachers. Each schoolteacher in Japan bears the weight of various responsibilities besides lessons in class, including moral guidance, counseling parents, being an advisor to school clubs after class, participating in activities to enhance educational effects, and preparation of reports. Therefore, teachers often feel distress about various areas, and thus, the mental health of Japanese schoolteachers is lower than that of general workers [1,2]. In addition, the number of Japanese public schoolteachers who are on leave of absence for mental illness is showing an almost consistent upward trend, such that schoolteachers on leave of absence for mental illness accounts for more than 60% of all schoolteachers on leave of absence for any illness [3]. Because poor mental health of teachers affects not only teachers themselves but also their students, this is a socially important problem. Previous studies that examined the relationship between poor mental health or burnout of teachers and various factors have identified less years of experience as a teacher and young age [4-7], insufficient social support [5,8,9], and stress management skills of the individual [10-12] as contributing factors. In particular, a study of young and inexperienced teachers revealed that stress management skills of the individual, rather than job-related stressors, were significantly correlated with mental health [13]. Therefore, it is presumed that policies for not only decreasing stressors but also enhancing stress management skills of teachers are necessary to improve the mental health of schoolteachers.

### Cognitive Behavioral Therapy and Cognitive Flexibility

A preceding study reported that cognitive behavioral therapy (CBT) is useful for enhancing stress management skills of workers [14]. CBT enhances cognitive flexibility, thereby challenging and replacing maladaptive thoughts with more balanced and adaptive thinking [15,16]. According to Dennis and Vander Wal, cognitive flexibility reflects the following 3 aspects: the tendency to perceive difficult situations as controllable, the ability to perceive multiple alternative explanations for life occurrences and human behavior, and the ability to generate multiple alternative solutions to difficult situations [17]. Previous studies in healthy subjects have found that individual differences in cognitive flexibility significantly and effectively predicted good performance on tasks that required carefulness [18], and cognitive flexibility was involved in fluent creativity and new ideas [19]. It has also been reported that persons with a high feeling of happiness have high cognitive flexibility and executed tasks of higher creativity [19]. Therefore, CBT may improve the performance of schoolteachers as well as their stress management skills by improving their cognitive flexibility.

### Effect of Low-Intensity Cognitive Behavioral Therapy

Low-intensity CBT, a program to provide the essence of CBT using simple measures including group education, use of the Internet, and books, is now spreading to use CBT in a wide range of scenarios beyond clinical treatment. It is also reported that stress management education using CBT performed on the Internet (Web-based CBT) alleviated the distresses of general workers and enhanced their cognitive flexibility [20,21]. However, there has been no study that examined whether training programs of schoolteachers using low-intensity CBT would enhance their cognitive flexibility and be effective for reducing their stress.

In this regard, this study provided schoolteachers with simple stress management training using a Web-based CBT program that allowed each of them to complete CBT homework independently, and investigated whether the training would contribute to the enhancement of cognitive flexibility and reduction of stress among teachers.

## Methods

### Participants

This study included 241 schoolteachers serving in public elementary schools in Sagamihara City in Kanagawa, Japan, who were in the fifth year of their career and were prospective participants in a mental health–related training program in 2014. We obtained permission from the board of education of Sagamihara City for the mental health training in 2014 to be performed as part of this study to evaluate the efficacy of a stress management training program using CBT. The board of education recommended that potential participants receive stress management training using CBT; however, there were no exclusion criteria set for study participants.

In a preceding study that used an intervention similar to ours in general workers [21], the effect size (Cohen d) for improvement of cognitive flexibility was 0.37. On the basis of this result, using an effect size of 0.37, an alpha error of .05, and a beta error of .20, the sample size required for this study was estimated to be 116 persons in each group to a total of 232 participants. Because the number of prospective participants in the training program in 2014 was 241, we considered that the necessary number of participants would be secured.

### Ethical Considerations

All potential study participants were instructed orally or in writing by the investigator before the beginning of the study that they could discontinue participation at any time if problems were caused by the intervention, and that this study was to be conducted by an organization independent of the board of education and would have no influence on personnel evaluation. Only the persons who gave consent to this study after this explanation were included in this study. They were also instructed that participation in the study should be completely of their own volition and that not participating in the study would cause no disadvantages. The study protocol was approved by the Ethics Committee of the School of Allied Health Sciences at Kitasato University. Reporting of methods and results of this study are based on the Consolidated Standards of Reporting Trials of Electronic and Mobile HEalth Applications and onLine TeleHealth (CONSORT-EHEALTH) guidelines [22]. This study was not registered because it is a pilot study.

### Procedure

The participants in the study were randomly allocated to 2 groups, that is, the intervention group and control group. All participants in the intervention group were given 1 session of group education and a Web-based CBT program lasting for 3 months. The efficacy of the intervention was evaluated based on the results of a self-administered questionnaire survey performed at baseline and 3 months after the completion of the CBT program. For ethical reasons, participants in the control group (waiting-list control) were also given the same group education and Web-based CBT program after the end of the study.

**Table 1:** The contents of the program.

Program and content		Points addressed
**Group CBT**^a^ **education (2 hours)**		
	Part 1. Lecture	The relationship between cognition, mood, and behavior
		What is CBT?
		Significance of learning CBT as a stress-coping method
	Part 2. Group work and discussion for learning cognitive restructuring	How to fulfill the column sheet?
		Recognize own inclination in the way of thinking
		Cooperate as a team in considering contrary evidence and adaptive thoughts
		Use of Web-based CBT program
Web-based homework using the Web-based CBT program		Delivering an email 6 times to urge implementation
		Implementation of at least 3 sessions recommended

^a^CBT: cognitive behavioral therapy.

### Intervention

The intervention consisted of 1 session of group education about CBT (lecture on CBT, group session using a column table), homework using the Web-based CBT program for the subsequent 3 months, and 6 emails sent during the self-learning period to stimulate the implementation of the Web-based CBT program (Table 1).

#### Group Cognitive Behavioral Therapy Education

A specialist in CBT took charge of the group education. One session of group education consisted of part 1 and part 2, taking 120 min in total. In addition, there were 60 participants in one session, and with the therapist was 1 leader (specialist in CBT), 1 coleader (specialist in CBT), and 3 assistants.

Part 1 was a seminar on the basic theory of CBT. Because the participants in this study were healthy, their awareness that CBT is useful for work and daily life was key to enhance motivation for education. Therefore, the purpose of including CBT in mental health education of teachers was clearly specified, such that CBT was not used as a treatment for mental illness but as a method of self-care to cope flexibly with various stress factors. The seminar was devised to use concrete examples and avoid technical jargon as far as possible to make the contents readily comprehensible.

Part 2 comprised group training in cognitive restructuring, the core technique of CBT. Cognitive restructuring helps people identify negative thought patterns, understand that these thoughts are ineffective or disruptive, and learn how to think differently by replacing adverse and illogical thoughts with more rational and adaptive types of thinking. In this part, participants learned about cognitive restructuring by recording the following on a worksheet about a recently experienced familiar (benign) situation: context, mood, automatic thinking, evidence, contrary evidence, adaptive thinking, and change of mood. During practice, participants were divided into groups of 5-7 members. In each group, one member described a recently experienced stressful event (a mild one causing no privacy issues) to the other members and his or her mood and automatic thinking at that time. Then, all members of the group considered evidence, contrary evidence, adaptive thinking, etc to practice completing the columns of the worksheet. Finally, each group presented the contents of the worksheet to all participants to share patterns and to ask a CBT specialist questions that arose during the group work to deepen their understanding of cognitive restructuring. At the end of the group training, the Web-based CBT program was introduced to participants so that they could learn how to manipulate the program.

#### Web-Based Homework Using the Web-Based Cognitive Behavioral Therapy Program

The homework using the Web-based CBT program lasted for 3 months after the end of the group education. The Web-based CBT program used *Mind Skill Up Training* [23]. This website, developed by a specialist in CBT, is designed for trainees to practice cognitive restructuring by themselves using the guide. This site is open to the public for use with a charge. Each participant in this study was given an ID and password by the investigator to access the website. Self-learning on the Web was feasible in the workplace or at home as long as a proper information technology environment was in place; the place of self-learning varied according to the convenience of the participant. During the homework implementation period, an email from a health nurse who was one of the investigators of this study was delivered to participants 6 times to provide information on CBT and urge them to do homework. Implementation of at least 3 sessions of homework was encouraged.

### Outcome Evaluation

The outcomes were cognitive flexibility and degree of subjective distress. Questions about these items were prepared, and evaluation was performed at baseline and 3 months after the end of the homework period, using self-administered questionnaires. Cognitive flexibility was evaluated in terms of the following 3 points proposed by Dennis and and Vander Wal: “I have a tendency to perceive difficult situations as controllable,” “I can perceive multiple alternative explanations for difficult situations,” and “I can generate multiple alternative solutions to difficult situations.” These items were rated according to 5 grades (1: not at all to 5: completely applicable). Subjective distress was examined by the question “How much do you perceive stress at work?” and rated according to 10 grades (1: not at all to 10: very strongly).

In addition, participants were examined for their base attributes, including depression, in terms of the Beck depression inventory, which is an index with established reliability and validity developed by Beck et al [24], and the degree of mental health based on the K6, which is a screening questionnaire with established validity and reliability developed by Kessler et al. This questionnaire comprised the following 6 items for screening the state of depression and mood or anxiety disorders in the general population [25]: overtime hours, hours of sleep, marital status, drinking habits, exercise habits, and history of seeking medical advice for mental illness as well as age, gender, and employee number.

### Randomization and Masking

An independent researcher who had no direct contact with the participants used computer-generated randomization with a 1:1 ratio and block size of 6. No stratification was performed and evaluators were masked. Owing to the nature of the intervention, participants were informed of their allocation status.

### Statistical Analysis

A generalized linear model was used for estimation, based on an intention-to-treat (ITT) analysis. To satisfy the ITT requirement that analyses be conducted for all participants, a multiple imputation (MI) method was used on the assumption that data could be considered missing at random. MI allows for uncertainty caused by missing data by generating several different plausible imputed datasets using a set of external covariates and appropriately combining results obtained from each [26,27].

The effect was calculated by subtracting the baseline outcome scores from those obtained after completion of 3 months of homework. Results are shown as changes in the raw scores for outcomes.

To analyze baseline characteristics of the study participants, a test was used for numerical variables and a chi-square test for categorical variables. Statistical significance was set at *P*<.05. IBM SPSS 22 and IBM SPSS Missing Values 22 (SPSS Inc., Chicago, IL, USA) were used for statistical analyses.

## Results

### Participants

**Figure 1:**
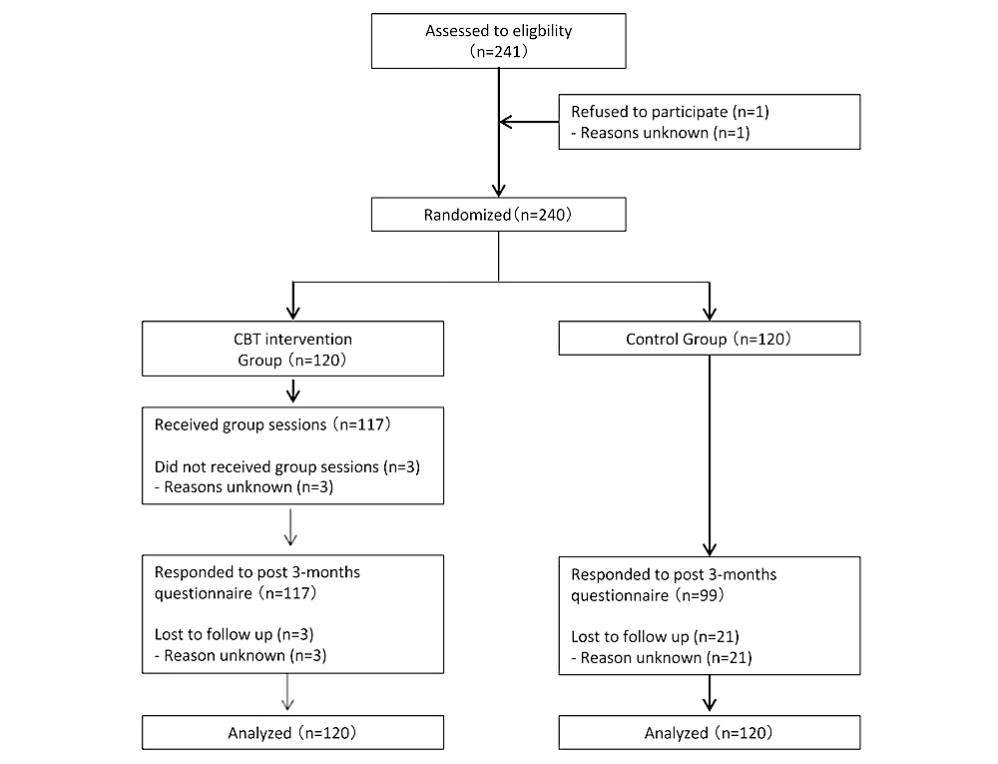
Study flowchart. CBT: cognitive behavioral therapy.

The flowchart of this study is shown in Figure 1. Among 241 participants who were prospective participants and who were in the fifth year of their career, 240, excluding 1 who refused participation, gave consent to the study.

**Table 2:** Baseline characteristics of the participants.

Characteristics		CBT^a^ group (N=120)	Control group (N=120)	Total (N=240)	*P* value
**Gender**					.74
	Male, n (%)	39 (32.5)	56 (46.7)	95 (38.9)	
	Female, n (%)	81 (67.5)	64 (53.3)	145 (41.2)	
Age in years, mean (SD)		29.9 (4.3)	30.5 (6.0)	30.3 (5.3)	.43
**Monthly mean overtime hours, n (%)**					.85
	Almost none	2 (1.8)	1 (1.1)	3 (1.5)	
	Less than 40 hours	25 (22.3)	19 (20.2)	44 (21.4)	
	40-79 hours	66 (58.9)	54 (57.4)	120 (58.3)	
	80 hours or more	19 (17.0)	20 (21.3)	39 (18.9)	
	Missing	8 (6.7)	26 (21.7)	34 (14.2)	
**Marital status, n (%)**					.16
	Unmarried	59 (52.2)	60 (62.5)	119 (56.9)	
	Married	54 (47.8)	36 (37.5)	90 (43.1)	
	No response	7 (5.8)	24 (20.0)	31 (12.9)	
**Drinking habits, n (%)**					.53
	Not at all	45 (39.8)	30 (31.6)	75 (36.1)	
	1-3 days a week	53 (46.9)	48 (50.5)	101 (48.6)	
	4-6 days a week	10 (8.8)	13 (13.7)	23 (11.1)	
	Every day	5 (4.4)	4 (4.2)	9 (4.3)	
	No response	7 (5.8)	25 (20.8)	32 (13.3)	
**Exercise habits, n (%)**					.67
	Not at all	46 (40.7)	43 (44.8)	89 (42.6)	
	1-2 days a week	58 (51.3)	48 (50.0)	106 (50.7)	
	3 or more days a week	9 (8.0)	5 (5.2)	14 (6.7)	
	No response	7 (5.8)	24 (20.0)	31 (12.9)	
**Mean weekday sleep time, n (%)**					.92
	Less than 5 hours	10 (8.8)	7 (7.3)	17 (8.1)	
	5-6 hours	77 (68.1)	66 (68.8)	143 (68.4)	
	7-8 hours	26 (23.0)	23 (24.0)	49 (23.4)	
	9 hours or more	0 (0.0)	0 (0.0)	0 (0.0)	
	No response	7 (5.8)	24 (20.0)	31 (12.9)	
**Mean weekend sleep time, n (%)**					.42
	Less than 5 hours	2 (1.8)	0 (0.0)	2 (1.0)	
	5-6 hours	19 (17.0)	16 (16.7)	35 (16.8)	
	7-8 hours	91 (81.3)	80 (83.3)	171 (82.2)	
	9 hours or more	0 (0.0)	0 (0.0)	0 (0.0)	
	No response	8 (6.7)	24 (20.0)	32 (13.3)	
**Poor mental health, n (%)**					.40
	Never sought medical advice	100 (89.3)	81 (84.4)	181 (87.0)	
	Sought medical advice once, but not seeing a doctor currently	11 (9.8)	12 (12.5)	23 (11.1)	
	Currently seeing a doctor regularly	1 (0.9)	3 (3.1)	4 (1.9)	
	No response	8 (6.7)	24 (20.0)	32 (13.3)	
K6, mean (SD)		3.24 (3.80)	3.83 (4.27)	3.51 (4.03)	.29
Tendency to perceive difficult situations as controllable, mean (SD)		3.14 (0.85)	3.12 (0.80)	3.13 (0.83)	.86
Ability to perceive multiple alternative explanations, mean (SD)		3.34 (0.84)	3.10 (0.86)	3.23 (0.85)	.04
Ability to generate multiple alternative solutions, mean (SD)		3.37 (0.91)	3.28 (0.89)	3.33 (0.90)	.48
Subjective distress, mean (SD)		6.03 (1.88)	6.28 (2.14)	6.14 (2.00)	.37

^a^CBT: cognitive behavioral therapy.

**Table 3:** Effect of intervention by the group cognitive behavioral therapy.

Variables		Intervention group, mean change (SE^a^)	Control group, mean change (SE)	Mean difference (95% CI)	*P* value	Effect size (d)
**Cognitive flexibility**						
	Tendency to perceive difficult situations as controllable	0.10 (0.08)	−0.19 (0.11)	0.29 (0.02-0.56)	.03	0.29
	Ability to perceive multiple alternative explanations	0.31 (0.10)	−0.26 (0.11)	0.57 (0.27-0.86)	<.01	0.52
	Ability to generate multiple alternative solutions	0.14 (0.12)	−0.15 (0.12)	0.29 (−0.03 to 0.60)	.08	0.24
Subjective psychological distress		−0.79 (0.24)	−0.13 (0.21)	0.66 (0.04-1.28)	.04	0.28

^a^SE: standard error.

The 240 participants were randomly divided into the intervention group and control group, each comprising 120 individuals. Of the 120 participants in the intervention group, 117 received group education. The remaining 3 who did not receive group education did not respond to the follow-up survey performed after 3 months of intervention. Reasons for the lack of response were not obtained, and the number of implementations of the Web-based program was unknown. In the control group, 99 participants responded to the follow-up survey after the intervention, whereas 21 did not (for reasons unknown). This study did not exacerbate any existing psychological problems of any participants.

### Baseline Characteristics

Table 2 shows baseline data. In regard to the male:female ratio of the participants, 39 (32.5%) of 120 individuals were men in the intervention group, whereas 56 (46.7%) were men in the control group. The mean age was 29.9 years (SD 4.3) in the intervention group and 30.5 years (SD 6.0) in the control group. There was no statistically significant difference in lifestyle or base attributes such as gender, age, years of service, position, overtime hours, hours of sleep, marital status, drinking habits, exercise habits, history of seeking medical advice for mental illness, and degree of mental health.

### Effects of the Training Program

Table 3 shows the effect of intervention by the group CBT. On comparing the scores before the intervention and after 3 months of the intervention, scores of outcomes related to cognitive flexibility, that is, “tendency to perceive difficult situations as controllable” and “ability to perceive multiple alternative explanations for difficult situations,” were significantly improved in the intervention group (mean difference 0.29 [95% CI 0.02-0.56], d=0.29 and mean difference 0.57 [95% CI 0.27-0.86], d=0.52, respectively). Although the outcome “ability to generate multiple alternative solutions to difficult situations” was also improved in the intervention group, the difference was not statistically significant (mean difference 0.29 [95% CI −0.03 to 0.60], d=0.24). In addition, the score for the degree of subjective distress was significantly decreased in the intervention group compared with the control group (mean difference 0.66 [95% CI 0.04-1.28], d=0.28).

## Discussion

### Principal Findings

The results of this study suggest that simple stress management training using CBT with elementary schoolteachers contributes to improvement of cognitive flexibility and reduction of subjective distress among these teachers.

Teachers are surrounded by diverse stress factors. Teachers in Japan must cope with various tasks and strive to solve problems of students and their parents. In the context of these diverse issues, it is suggested that reducing the risk of burnout of teachers requires teachers to recognize problems correctly and respond appropriately to a situation. Namely, it is desirable to acquire realistic and adaptive stress-coping behaviors based on high cognitive flexibility [10-12]. Stated differently, cognitive flexibility is the ability or tendency to abandon an unhelpful cognitive strategy and choose a different one when experiencing a problem that is difficult to fix [28]. This ability is indispensable in stress management of teachers who are often faced with student- or parent-related issues that are difficult to resolve. It has also been pointed out that cognitive flexibility contributes to not only prevention of burnout but also nurturing of prompt judgment for difficult tasks and improvement of the ability to recognize even a slight environmental change and to cope with it [18,29]. Teachers are also required to have the ability to promptly cope with consecutive daily problems and to quickly notice and react to changes in the atmosphere of students and the class. Therefore, teachers who have high cognitive flexibility may prevent a problem from growing through such coping ability, thereby reducing the number of stressors they face. Thus, improvement of cognitive flexibility seems important in the enhancement of stress management skills of teachers.

In this study, greater importance was placed on provision of a feasible program for busy teachers. Regarding mental health measures in the occupational field, previous intervention studies using CBT for individuals or groups have shown beneficial effects such as alleviation of depression or anxiety, reduction of stress [30-32], and improvement of absenteeism [30]. It has also been reported that CBT has a favorable effect on positive dimensions of mental health, such as improvement of the quality of work [30], quality of life [31], work functioning [26], and performance [30,31]. However, intervention programs used in these studies require a lot of time and continuous involvement of CBT specialists, and are, therefore, difficult to implement in the workplace in many cases. In this regard, recent years have seen the implementation of efficacy studies using a simplified CBT program by Internet, email, or telephone in the occupational field. However, most of these programs required much time to complete or frequent exchange of emails or telephone calls [32-37]. This study provided a very simple program consisting of 120-min group education and at least 3 sessions of homework (each session required a run-time of about 30 min) based on a simple Web-based CBT program. This was because we gave importance to the feasibility of the program for a greater number of healthy teachers while minimizing harm to work.

In addition, this study used some mechanisms to maintain motivation for training. Because CBT intervention in healthy subjects may be associated with low motivation for intervention, unlike patients who seek treatment, we emphasized to participants that training based on the principles of CBT in group education would be useful for coping with work stress and for improvement of productivity [38,39]. It has also been reported that reminding increases the implementation rate when CBT is performed on the Internet [40]. Therefore, 6 emails from a health nurse to urge completion of homework were designed to be delivered to each participant during the 3 months of homework. Furthermore, to raise the implementation rate, group education was provided during working hours, and homework was made feasible at home as well as in the workplace. Although the implementation rate of homework was not determined in this study, we presume that these mechanisms contributed to improvement of the implementation rate of homework.

### Limitations

This study had some limitations. First, evaluation indices were our original inquiry items. Although validated scales that evaluate psychological stress are present, and a limited number of scales for cognitive flexibility have been proposed, all these scales include many items. For this study, we chose to use simple questionnaire scales at the stage of study planning. Due to this, a 1-item scale was used for each outcome. Second, participants were limited to teachers in a city who were in the fifth year of their career. To increase the general validity of this study, this issue should be studied in a randomized controlled trial (RCT) design under wider conditions. Third, homework implementation status was not evaluated. This was aimed at reducing burden on the participants. However, because homework is considered to play an important part in CBT, it would have been desirable if we had analyzed in detail how often the participants performed the simplified CBT program and whether and how closely the number of implemented homework sessions was correlated with the effect of CBT. Fourth, evaluation of outcomes was completed only at 3 months after the completion of intervention. The decision to do this was made at the stage of study planning to reduce burden on the participants. However, longer follow-up would have been desirable, considering that the amount of work varies according to the time of year, and for the purpose of examining the long-term effect.

### Conclusions

This study was the first to examine whether an intervention using CBT in teachers would be effective for enhancement of the cognitive flexibility of teachers and alleviation of their subjective distress. The results of this study show that a simple intervention using Web-based CBT enhanced the cognitive flexibility of teachers and alleviated their subjective distress. Because the subjects of this study were busy schoolteachers, the methods of intervention and evaluation were simplified to the maximum possible extent. Therefore, this study had some limitations in its general validity, but it is of major significance that a useful means for stress management of teachers has been proposed. It is desirable that more useful programs using interventions based on CBT be developed and that a number of RCTs be performed to evaluate their effects appropriately.

## Appendix

Multimedia Appendix 1 CONSORT-EHEALTH checklist (V 1.6.1).

## Abbreviations

CBT: cognitive behavioral therapy
ITT: intention-to-treat
MI: multiple imputation
RCT: randomized controlled trial
